# Association of Preoperative Patient Resilience With Postoperative Patient-Reported Outcomes and Sleep Quality Following Arthroscopic Rotator Cuff Repair: A Systematic Review

**DOI:** 10.7759/cureus.60462

**Published:** 2024-05-16

**Authors:** Muzammil Akhtar, Sonia Aamer, Meraj Alam, Nabeal Dean, Lena Bondogji, Madina Tokhi, Shaheryar Asad, Daniel I Razick, Leeann Qubain, Suhair Bhatti

**Affiliations:** 1 Orthopedic Surgery, California Northstate University College of Medicine, Elk Grove, USA; 2 Psychiatry, California Northstate University College of Medicine, Elk Grove, USA; 3 Internal Medicine, California Northstate University College of Medicine, Elk Grove, USA; 4 Surgery, California Northstate University College of Medicine, Elk Grove, USA; 5 Orthopedic Surgery, University of Arizona College of Medicine-Phoenix, Phoenix, USA; 6 Psychiatry, St. Joseph’s Medical Center, Stockton, USA

**Keywords:** brief resilience scale, outcomes, resilience, arthroscopic, rotator cuff repair

## Abstract

Recent studies have shown that low preoperative resilience may lead to inferior outcomes following arthroscopic rotator cuff repair. Therefore, the purpose of this systematic review is to evaluate whether preoperative patient resilience is associated with outcome measures, including patient-reported outcome measurements (PROMs) and sleep quality, following arthroscopic rotator cuff repair. To perform the review, a literature search was conducted according to Preferred Reporting Items for Systematic Reviews and Meta-Analyses (PRISMA) guidelines using the PubMed and Embase databases to gather studies related to the influence of preoperative resilience on postoperative outcomes of rotator cuff repair. Methodological quality and risk of bias were assessed using the Methodological Index for Non-randomized Studies (MINORS). Seven studies with 584 patients were included. Of 36 total reported postoperative outcomes, including PROMs and sleep quality, 14 had a significant positive correlation with higher preoperative resilience. One study reported that higher resilience was significantly correlated with worse sleep quality at a two-week follow-up but not at further follow-ups of up to 24 weeks. Significant differences in outcomes between patients with varying levels of resilience were assessed in five studies, all of which found that patients with higher resilience had significantly better outcomes or no significant differences in outcomes between patients with varying levels of preoperative resilience. In no study was it reported that patients with low resilience had better outcomes. Overall, approximately half of all reported postoperative outcome data was found to be significantly associated with preoperative resilience. Therefore, clinicians should preemptively identify those with low resilience and administer psychological interventions to limit inferior outcomes following arthroscopic rotator cuff repair.

## Introduction and background

Rotator cuff tears are prevalent in 8.2 to 23.0% of the population and are the most common cause of shoulder-related disability, with their etiology being multifactorial combinations of factors such as age-related degenerative changes, trauma, smoking, hypercholesterolemia, and family history [1-7]. Rehabilitation and physical therapy have been demonstrated to be an effective conservative treatment with the most favorable outcomes particularly observed in partial-thickness, degenerative nontraumatic full-thickness, and massive irreparable tears [8]. Surgical repair is often indicated for acute partial-thickness tears, full-thickness tears in younger patients, or full-thickness tears in older patients who have failed initial nonoperative treatment [9]. Though historically repaired using an open approach, surgical and technological advancements have allowed the adoption of arthroscopy to minimize complications associated with open repairs, such as poor visualization, risk of infection, and greater pain [9].

Historically, factors associated with outcomes of rotator cuff repair surgery have included tear size, patient age, baseline patient-reported outcome measurement (PROM) scores, fatty infiltration, muscle atrophy, comorbidities, and surgical technique [10-11]. However, studies have additionally supported a biopsychosocial model in which psychosocial factors such as anxiety, depression, and fear avoidance beliefs have been found to be significantly associated with function, disability, and pain following rotator cuff repairs [12-13]. While emotional distress and ineffective coping strategies have been demonstrated to contribute to functional limitations and pain, a more recently researched topic has been the association between positive psychology, defined as constructs that enable individuals to thrive and adapt to challenges and surgical outcomes. Positive psychology factors include satisfaction with life, gratitude, coping through humor, resilience, mindfulness, and optimism [14]. Resilience, defined as the ability to bounce back from stress or injury, is a specific aspect of positive psychology that has been investigated across multiple areas within orthopedic surgery including total joint arthroplasty, anterior cruciate ligament reconstruction, spine surgery, foot and ankle surgery, and upper extremity surgery, with varying conclusions regarding the association between preoperative resilience and postoperative outcomes [15-20].

There is similarly no clear consensus within the literature regarding the association between preoperative resilience and postoperative outcomes of arthroscopic repair of rotator cuff tears. Therefore, the purpose of this systematic review is to evaluate whether preoperative patient resilience is associated with outcome measures, including PROMs and sleep quality, following arthroscopic rotator cuff repair. We hypothesized that patients with higher preoperative resilience would demonstrate better postoperative outcomes compared to those with lower resilience.

## Review

Methods

Search Strategy

A search following guidelines established by the Preferred Reporting Items for Systematic Reviews and Meta-Analyses (PRISMA) was performed in two databases on December 12, 2023: PubMed and Embase. The following search strategy was used to perform the systematic review: resilience AND (shoulder OR rotator cuff OR glenoid OR glenohumeral OR labrum OR labral OR arthroscopy). There were no limitations to our search. 

Study Selection

Two independent reviewers reviewed studies for eligibility criteria from the initial database search. A third reviewer was available for any disputes. Studies were included if they evaluated the relationship between preoperative patient resilience and postoperative outcomes of arthroscopic rotator cuff repair. Exclusion criteria included case reports, review articles, cadaveric studies, articles not in English, expert opinions, open surgery, conference abstracts, biomechanical studies, and editorial commentaries. All included articles underwent a rigorous review of their reference lists to determine whether additional studies could be added to the systematic review based on our inclusion criteria.

Data Extraction

Study variables extracted from the studies included author, publication year, journal, level of evidence (LOE), number of patients, rotator cuff tear characteristics, patient sex, mean follow-up time, mean age, preoperative and postoperative outcomes stratified by patient resilience, and correlations between patient resilience and outcomes after arthroscopic rotator cuff repair. All extracted data was compiled for analysis using Microsoft Word (Microsoft Office 2011; Microsoft, Redmond, WA).

Quality Assessment and Risk of Bias

The risk of bias and methodological quality were assessed using the Methodological Index for Non-randomized Studies (MINORS) score. The MINORS criteria are scored 0 (not reported), 1 (reported but inadequate), or 2 (reported and adequate), with a maximum possible score of 16 for noncomparative studies and 24 for comparative studies. Noncomparative studies with MINORS scores of <8, 8-12, and 12-16 were determined to have a high, moderate, and low risk of bias, respectively. Comparative studies with MINORS scores of <16, 16-20, and 20-24 were determined to have a high, moderate, and low risk of bias, respectively.

Data Analysis

Descriptive statistics, including mean, percentage, standard deviation, range, median, 95% confidence intervals, and correlation coefficients are reported in this review when applicable and when available. A meta-analysis was performed using a random effects model to compare PROMs between patients with high versus low resilience. The meta-analysis was performed only if the same PROM was present in three or more studies. Forest plots were generated using Cochrane’s Reviewer Manager web application (RevMan; computer program, version 5.4, The Cochrane Collaboration, 2020). A P-value less than 0.05 was considered statistically significant.

Results

Article Selection Process

**Figure 1:**
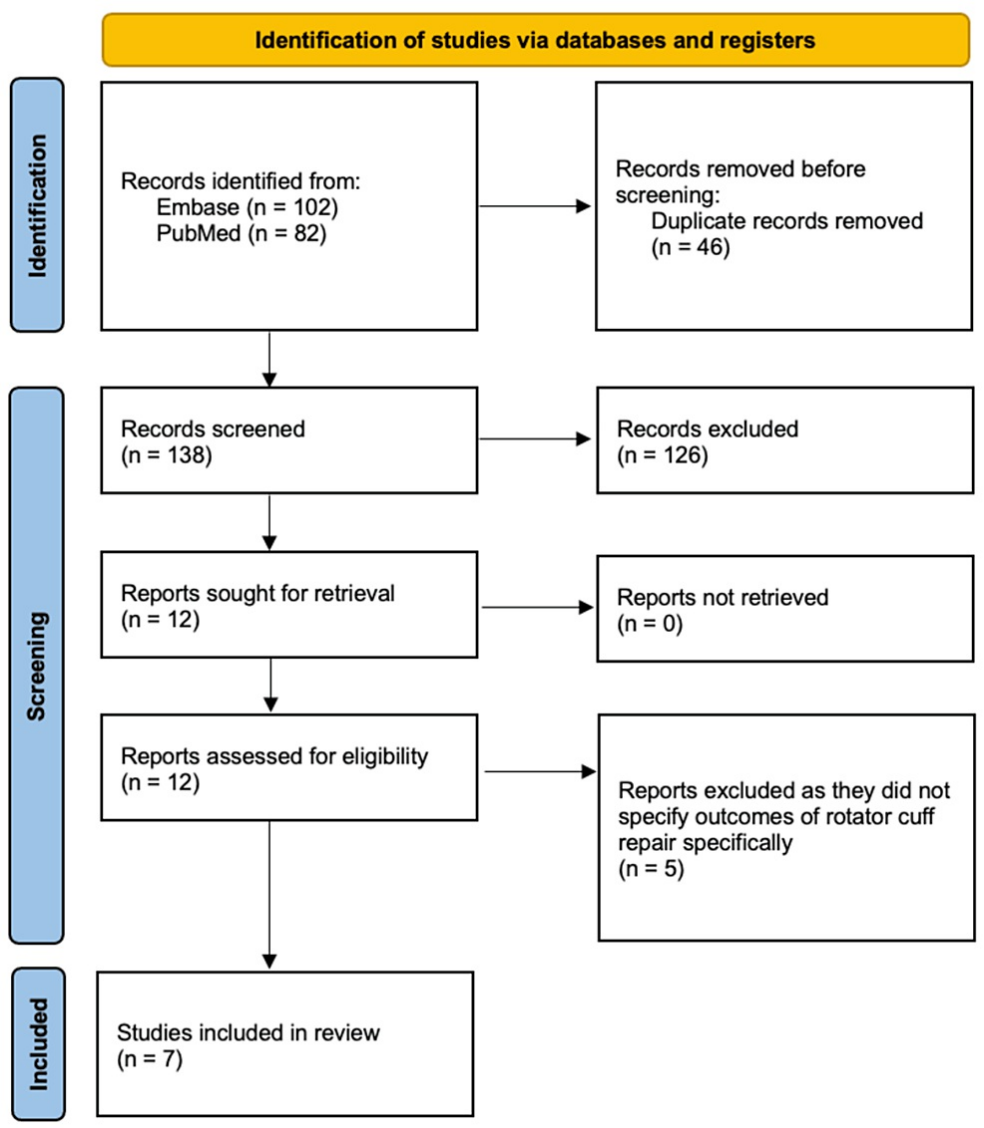
PRISMA flow diagram depicting the article selection process. PRISMA, Preferred Reporting Items for Systematic Reviews and Meta-Analyses.

Upon the initial search of the PubMed and Embase databases, 184 studies were identified, of which 46 duplicates were removed. The remaining 138 studies underwent full title and abstract review, of which 126 were removed based on our predetermined exclusion criteria. The remaining 12 studies underwent full-text review to determine whether they met our inclusion criteria, and five were excluded as they did not specify outcomes of rotator cuff repair alone. Seven studies were therefore included in this systematic review [21-27]. The PRISMA flow diagram depicting our search strategy and method of selecting articles is depicted in Figure 1.

Methodological Assessment and Risk of Bias

The mean ± standard deviation of the MINORS score for the seven studies was 12.6 ± 1.8 (range, 10 to 15). Four studies were deemed to have a low risk of bias, whereas three studies were deemed to have a moderate risk of bias. No study had a high risk of bias.

Study Characteristics and Demographic Data

Three studies each had a LOE of II and IV, and one study had a LOE of III. Five studies used the Brief Resilience Scale (BRS), one used the Revised Life Orientation Test (LOT-R), and another used the Connor Davidson Resilience Scale (CD-RISC). Three studies specified the range of scores on these resilience scales to determine whether a patient had low, normal, or high resilience. Across all studies, there were a total of 584 patients (52.4% male and 47.6% female). Three studies, comprising 308 patients, reported the tear size, with 60.4% of patients having small tears (<3 cm) and 39.6% of patients having large tears (>3 cm). Two studies, comprising 106 patients, reported whether tears were partial or full-thickness, with 26.4% having partial-thickness tears and 73.6% having full-thickness tears. The pooled mean age of all patients was 60.0 years and follow-up times ranged from three to 48 months. 

Tracy et al. reported that patients with low versus high resiliency, based on the BRS, were not significantly different in any demographic variable, including age, sex, tobacco use, worker’s compensation status, tear characteristics, and number of anchors used (P = 0.149 to > 0.999) [25]. Petrie et al. also found that patients with low versus high resiliency, based on the BRS, were not significantly different in any demographic variable, including age, BMI, symptom duration, patient, previous surgical procedures, and current narcotic pain medication use (P = 0.090 to 0.933) [23]. Table 1 summarizes the methodological assessment, study characteristics, and demographic data for all included studies.

**Table 1:** Study characteristics and patient demographic information. The resilience measurement column reports the questionnaire administered to patients to evaluate resilience. Three studies provided ranges of scores that categorized patients as having a certain level of preoperative resilience [21-27]. LOE, level of evidence; MINORS, Methodological Index for Non-randomized Studies; BRS, Brief Resilience Score; LOT-R, Revised Life Orientation Test; CD-RISC, Connor Davidson Resilience Scale; M/F, Male/Female; NR, not reported.

Author	LOE	MINORS	Resilience measurement	Patients (M/F)	Tear characteristics	Age in years	Follow-up
Wilson et al. [26]	II	15	BRS	98 (45/53)	Small (<1 cm): 27	60.8 (26-80)	3 and 6 months
					Medium (1 to <3 cm): 32		
					Large (3 to <5 cm): 14		
					Massive (≥5 cm): 25		
Wilson et al. [27]	II	13	BRS	91 (41/50)	Small (<1 cm): 23	61.6 (38-80)	32 (24-47) months
					Medium (1 to <3 cm): 29		
					Large (3 to <5 cm): 14		
					Massive (≥5 cm): 25		
Tracy et al. [25]	IV	11	BRS	Total: 57 (39/18)	Partial thickness: 6	62.5 (42-78)	34.5 months
			Low: <20.8	Low resilience: 9 (5/4)			
			Normal: 20.8-28.8	Normal resilience: 36 (25/11)	Full thickness: 51		
			High: >28.8	High resilience: 12 (9/3)			
Petrie et al. [23]	II	13	BRS	Total: 131 (57/74)	NR	57.6 ± 9.8	12 and 24 months
			Low: 14-19	Low resilience: 20 (9/11)			
			Normal: 20-28	Normal resilience: 85 (36/49)			
			High: 29-30	High resilience: 26 (12/14)			
Hines et al. [22]	III	10	BRS	110 (71/39)	Small (≤3 cm): 75 (43/32)	61 ± 10	12 months
					Large (>3 cm): 44 (28/16)		
Porter et al. [24]	IV	11	LOT-R	Total: 49 (28/21)	Partial thickness: 22	55 ± 7.9 (32-67)	48 months
								Low: 0-6	Low resilience: 0
			Mild: 7-12	Mild resilience: 5					
								Full thickness: 27
			Moderate: 13-18	Moderate resilience: 25				
					High: 19-24				High Resilience: 19
			Glogovac et al. [21]	IV						15	CD-RISC	48 (25/23)	NR	62 (34-81)	6 months

Differences Between Patients With Varying Levels of Resilience

Wilson et al. found that of patients with (n = 43) versus without (n = 55) depression or anxiety there were no differences in preoperative (22.5 versus 24.3, P = 0.303), three-month (22.7 versus 24.7, P = 0.252) or six-month (21.7 versus 24.3, P = 0.166) BRS scores [26]. Tracy et al. stratified patients into low, normal, and high resiliency groups based on preoperative BRS scores and compared the low and high resiliency groups [25]. The preoperative Single Assessment Numeric Evaluation (SANE) score was significantly higher in the high resiliency group (38.5 versus 19.6, P = 0.026), as was the postoperative American Shoulder and Elbow Surgeons (ASES) score (90.2 versus 68.5, P = 0.031). Preoperative range of motion (ROM), Visual Analogue Scale (VAS) pain, ASES, and VR-12 scores, along with two-year follow-up ROM, VAS pain, SANE, VR-12, satisfaction, and return to work/sport, were similar between low and high resiliency groups. Petrie et al. stratified patients into low, normal, and high resiliency groups based on preoperative BRS as well [23]. The VR-12M score was significantly higher in the high versus low and normal resiliency groups at both 12 and 24 months (P = 0.0002 and 0.0014, respectively), whereas all three resilience groups showed no significant differences in 12-month and 24-month follow-up ASES, SANE, Simple Shoulder Test (SST), VAS pain, and VR-12P scores (P = 0.069 to 0.931). Hines et al. compared preoperative BRS scores between patients who did versus those who did not meet the significant clinical benefit (SCB) threshold for the ASES score at a six-month follow-up [22]. They found there to be no differences in preoperative BRS scores between those who did versus those who did not meet the SCB threshold (23.5 versus 23.5, P = 0.97). Similarly, they found there to be no difference between BRS scores and large or small rotator cuff tear size (P = 0.12).

Porter et al. stratified patients into low, mild, moderate, and high resiliency groups based on the four-year follow-up LOT-R score; no patient had low resiliency [24]. The high and moderate groups had significantly higher ASES (90.7 and 85.8 versus 62.0, P = 0.005) and SST scores (10.8 and 10.4 versus 6.8, P = 0.009) compared to the mild group, whereas there were no significant differences between scores in the high versus moderate groups.

**Figure 2:**
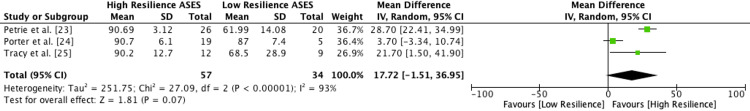
Forest plot depicting the postoperative ASES score in patients with high versus low preoperative resilience. ASES, American Shoulder and Elbow Surgeons.

A meta-analysis of three studies found that though patients with high resilience had higher postoperative ASES scores, there were no significant differences in ASES scores between high versus low resilience patients (Mean Difference: 17.72; 95% Confidence Interval: -1.51 to 26.95; P = 0.07; Figure 2).

Correlations Between Resilience and Outcome Measures

Wilson et al. found that preoperative BRS scores were positively and significantly correlated with only three-month Patient-Reported Outcomes Measurement Information System (PROMIS)-10 scores (r = 0.38, P = 0.009) [26]. There were no correlations between preoperative BRS scores and three-month ASES, VAS pain, and SANE scores, along with six-month ASES, PROMIS-10, VAS pain, and SANE scores (r = -0.16 to 0.24, P = 0.262 to 0.831). A significant correlation was also found between three-month BRS and three-month PROMIS-10 scores (r = 0.57, P = 0.0025), along with six-month BRS and six-month PROMIS 10 scores (r = 0.53, P = 0.0025). In their later study, Wilson et al. assessed the correlation between both preoperative and postoperative resilience scores, BRS in this case, with PROMs at a 2.7-year follow-up [27]. Postoperative BRS compared to preoperative BRS scores had a much stronger correlation with postoperative ASES (r = 0.29 versus 0.16, P = 0.005 versus 0.142), PROMIS-10 (r = 0.52 versus 0.29, P < 0.001 versus = 0.005), and SANE (r = 0.38 versus 0.26, P < 0.001 versus = 0.014) scores. Tracy et al. reported that regression analysis revealed a significant association between BRS and two-year ASES score, as with every 1-point increase in the BRS score, the ASES score increased by 1.6 points (P = 0.012) [25]. Petrie et al. found significant positive correlations between preoperative BRS scores and 12-month SST (r = 0.18), VR-12P (r = 0.21), VR-12M (r = 0.38), and 24-month VR-12M (r = 0.31) [23]. There were however no significant correlations between the preoperative BRS score and VAS pain, ASES, and SANE scores at 12 and 24 months postoperatively.

Porter et al. found a significant positive correlation between LOT-R scores with ASES (P = 0.005) and SST (P = 0.018) scores, whereas past or present tobacco use, alcohol abuse, and patient sex had no significant correlation with ASES (P = 0.111 to 0.367) and SST (P = 0.270 to 0.773) scores [24].

Glogovac et al. aimed to determine whether resilience, as measured with the CD-RISC, was related to nocturnal pain frequency and sleep quality, as measured with the PQSI, at 2, 6, 12, and 24 weeks following arthroscopic rotator cuff repair [21]. They found a significant positive correlation between the CD-RISC and nocturnal pain frequency (r = 0.28, P = 0.041) and Pittsburgh Sleep Quality Index (PSQI; r = 0.30, P = 0.028) at only the two-week mark, indicating worse sleep quality in those with higher resilience; beyond that, there were no significant correlations at any other time point.

Table 2 summarizes the correlations between resilience and outcome measures along with additional significantly different findings between patients with varying levels of resilience.

**Table 2:** Correlations between postoperative outcomes and patient resilience. Resilience scores are reported as mean ± standard deviation (range) when available [21-27]. BRS, Brief Resilience Score; LOT-R, Revised Life Orientation Test; CD-RISC, Connor Davidson Resilience Scale; ASES, American Shoulder and Elbow Surgeons Score; PROMIS-10, Patient-Reported Outcomes Measurement Information System-10; VAS, Visual Analog Scale; SANE, Single Assessment Numeric Evaluation; SST, Simple Shoulder Test; VR-12M, Veterans RAND 12 Item Health Survey Mental Component; VR-12P, Veterans RAND 12 Item Health Survey Physical Component; PQSI, Pittsburgh Sleep Quality Index; NR, not reported.

Author	Resilience measure		Correlations with resilience			Additional significant findings
	Measure	Score	Outcome measure at follow-up	Correlation coefficient	P-value	
Wilson et al. [26]	BRS	Preoperative: 23.5 (12-30)	3-month ASES	0.04	0.831	NR
			3-month PROMIS-10	0.39	0.009	
			3-month VAS pain	-0.06	0.770	
			3-month SANE	0.11	0.664	
			6-month ASES	0.12	0.627	
			6-month PROMIS-10	0.24	0.262	
			6-month VAS pain	-0.16	0.502	
			6-month SANE	0.07	0.754	
Wilson et al. [27]	BRS	Preoperative: 23.5 (12-30)	ASES	0.16	0.142	NR
			ASES pain	0.07	0.520	
			ASES functional	0.22	0.035	
			PROMIS-10	0.29	0.005	
			PROMIS-10 physical	0.08	0.444	
			PROMIS-10 mental	0.29	0.005	
			SANE	0.26	0.014	
		32-month follow-up: 23.8 (11-30)	ASES	0.29	0.005	
			ASES pain	0.24	0.036	
			ASES functional	0.29	0.006	
			PROMIS-10	0.52	< 0.001	
			PROMIS-10 physical	0.25	0.019	
			PROMIS-10 mental	0.50	< 0.001	
			SANE	0.38	< 0.001	
Tracy et al. [25]	BRS	Preoperative: 24.8 ± 4	ASES	NR	0.012	Significantly higher preoperative SANE scores in patients with both High versus Low preoperative resilience scores (P = 0.026). Significantly higher postoperative ASES scores in patients with High versus Low preoperative resilience scores (P = 0.031)
Petrie et al. [23]	BRS	Preoperative: 24 ± 4.2	12-month ASES	0.15	> 0.05	Significantly higher preoperative VR-12M scores in patients with High and Normal versus Low preoperative resilience scores (P = 0.001). Significantly higher postoperative VR-12M scores in patients with High and Normal versus Low preoperative resilience scores (P = 0.001)
			12-mont ASES functional	0.13	> 0.05	
			12-month VAS pain	-0.15	> 0.05	
			12-month SANE	0.12	> 0.05	
			12-month SST	0.18	< 0.05	
			12-month VR-12M	0.38	< 0.05	
			12-month VR-12P	0.21	< 0.05	
			24-month ASES	0.07	> 0.05	
			24-month ASES functional	0.09	> 0.05	
			24-month VAS pain	-0.04	> 0.05	
			24-month SANE	0.00	> 0.05	
			24-month SST	0.12	> 0.05	
			24-month VR-12M	0.31	< 0.05	
			24-month VR-12P	0.12	> 0.05	
Hines et al. [22]	BRS	Preoperative: 23.5	NR	NR	NR	No postoperative outcome differences between patients with varying tiers of preoperative resilience
Porter et al. [24]	LOT-R	NR	ASES	0.13	0.005	Significantly higher postoperative ASES, ASES functional, ASES pain, and SST in High and Moderate versus Mild preoperative resilience scores (P = 0.003 to 0.048)
			ASES functional	0.31	0.029	
			ASES pain	0.42	0.003	
			SST	0.19	0.018	
Glogovac et al. [21]	CD-RISC	NR	2-week PQSI	0.30	0.028	NR
			2-week nocturnal pain frequency	0.28	0.041	

Discussion

Primary Findings

The primary purpose of this systematic review was to determine whether patient resilience was associated with outcome measures following arthroscopic rotator cuff repair. Across all seven studies, there was a significant positive correlation between preoperative resilience and outcome measures in 14 of 36 total reported outcome measures at various postoperative time points. No study reported significant negative correlations between preoperative resilience and postoperative outcomes. Significant differences in postoperative outcomes between patients with varying levels of preoperative resilience were assessed in five studies. In all five studies, patients with higher preoperative resilience were found to have significantly better postoperative outcomes, or there were no significant differences in outcomes in patients with varying levels of preoperative resilience. In no study was it reported that patients with low resilience had better postoperative outcomes. Overall, approximately half of all reported postoperative outcome data was found to be significantly associated with preoperative resilience. However, there appeared to be no apparent patterns between preoperative resilience and time of postoperative outcome measurement or specific outcome measurement assessed, such as the ASES and SANE scores.

Two of the most assessed postoperative outcomes were the ASES and SANE scores. Only two of five studies found a significant positive correlation between resilience and postoperative ASES. Interestingly, both of these studies recorded the ASES score at the longest follow-up of any study at 34.5 and 48 months, respectively. Of four studies assessing differences in ASES scores based on varying levels of resilience, two found there to be higher ASES scores in patients with higher resilience, one found there to be no difference in ASES scores, and one found there to be no difference in resilience between patients who did versus did not achieve the SCB threshold for the ASES score. Only one of three studies found a significant positive correlation between resilience and postoperative SANE scores. Of two studies assessing differences in SANE scores based on varying levels of resilience, only one found there to be significantly higher scores in patients with higher resilience, whereas the other study found there to be no difference.

While six of seven studies measured postoperative outcomes as patient-reported outcomes, Glogovac et al. measured outcomes based on postoperative sleep quality as measured with the PSQI and nocturnal pain frequency [21]. At a postoperative follow-up period of up to six months, they found that higher preoperative resiliency was significantly and positively correlated with worse sleep quality outcome measures at only the two-week mark. This is a counterintuitive finding as one would expect the opposite where those with higher resilience had better sleep quality. It is unclear as to why this occurred; however, the entire patient cohort, regardless of resilience level, had the worst sleep quality at the two-week mark, which contradicts other studies which report that the worst sleep quality is before rotator cuff repair [28]. Poor sleep quality is known to be associated with rotator cuff tears, with arthroscopic repair being an effective treatment to improve sleep quality [29]. A systematic review found that consistent improvements in sleep quality were observed in the six months following arthroscopic rotator cuff repair, yet some patients still had a high burden of poor sleep [29]. Therefore, though the study by Glogovac et al. was the first of its kind, future studies should aim to determine whether there is a relationship between preoperative resilience and postoperative sleep quality to preemptively identify patients at risk of inferior outcomes [21].

Scales Measuring Resilience

Most studies were limited to a short-term follow-up during which relationships between preoperative resilience and outcomes at follow-up were assessed. We noted an interesting finding in the study by Porter et al. in which at a four-year follow-up, the longest of any study, all patient-reported outcomes, including the ASES, ASES functional component, ASES pain component, and SST scores, all showed a significant positive correlation with preoperative resilience [24]. However, Porter et al. used the LOT-R scale to assess resilience, whereas most studies commonly use the BRS or CD-RISC [24]. The LOT-R scale quantifies resiliency by assigning a value to optimism or pessimism, with the idea that optimism correlates directly with resiliency. To the best of our knowledge, no studies evaluating the relationship between resiliency and postoperative outcomes have used the LOT-R scale, but have rather used the BRS or CD-RISC scales which may better be suited to directly assess resiliency in orthopedic surgery [15,22-23,25-27,30-35]. While both the CD-RISC and BRS scales are used to assess a patient’s ability to bounce back from stressful events, they can capture various aspects of resilience. The CD-RISC emphasizes the resources available for individuals to recover from and adapt to disruptions or stress, while the BRS directly measures a patient's resilience or ability to bounce back. Though the results of Porter et al. are optimistic, especially as they provide data with longer-term follow-up, their use of the LOT-R scale serves as a limitation [24]. Future studies should use both the LOT-R and BRS scales preoperatively and then assess whether both scales are similarly related to postoperative outcomes.

Psychological Interventions to Improve Resilience

Since there were significant associations between higher preoperative resilience and better postoperative outcome measures, it may be beneficial to identify patients with low resilience and administer preoperative resilience training through psychological interventions to help improve postoperative outcomes. A recent meta-analysis of randomized controlled trials evaluated cognitive behavioral therapy (CBT) based, mindfulness-based, and mixed CBT and mindfulness-based interventions to improve resilience [36]. The authors of this study found the standard mean difference (SMD) between CBT-based, mindfulness-based, and mixed interventions versus control groups to be 0.27, 0.46, and 0.51, respectively, indicating a positive low to moderate effect. CBT is a therapeutic approach aimed at assisting individuals in recognizing and altering harmful thought patterns that adversely impact their actions and feelings. This fosters a more balanced mindset, enhancing the capacity to manage stress effectively [37]. A model to build resilience via CBT was proposed in 2012, which included four steps: (1) search for strengths, (2) construct a personal model of resilience, (3) apply the personal model to areas of life difficulty, and (4) practice resilience. Mindfulness-based interventions, on the other hand, use methods such as meditation, relaxation, and awareness exercises to improve the ability to pay full attention to the present-moment experience without elaboration, judgment, or emotional reactivity [38]. The successful use of mindfulness-based preoperative therapy before total joint arthroplasty has successfully been reported in a recent randomized controlled trial. In the study, those who underwent mindfulness meditation had a significantly better six-week physical function and lower pain intensity, pain unpleasantness, medication desire, and anxiety compared to those who underwent hypnosis or cognitive behavioral pain psychoeducation. However, the effect of the three psychological interventions on resilience was not assessed in this trial [39].

Limitations

This systematic review should be considered in the context of its limitations. The total number of included studies was small which limits the generalizability of the conclusions. Though there were some similarities between studies in outcome measures reported, such as the ASES and SANE scores, most reported outcomes were different among the studies which limits our ability to conclude whether preoperative resilience is correlated to a specific outcome measure across the literature. Additionally, studies had varying follow-up periods which may have influenced the results. Though two studies did report correlations between resilience and outcomes at two different postoperative time points, we were unable to conclude whether longer follow-up periods strengthen or weaken the correlations. Future studies should aim to evaluate correlations between resilience and outcomes in a serial manner to determine whether time has an impact on the results. There was also variation in which scale was used to evaluate resilience, as five studies used the BRS, one used the LOT-R, and one used the CD-RISC. Though the BRS and CD-RISC are relatively similar, the LOT-R, to the best of our knowledge, has not been applied to evaluate resiliency in studies related to orthopedic surgery. 

## Conclusions

Approximately half of all reported postoperative outcome data was found to be significantly associated with preoperative resilience. There appeared to be no apparent patterns between preoperative resilience and time of postoperative outcome measurement or specific outcome measurement assessed. However, clinicians should consider preemptively identifying those with low resilience and recommend psychological interventions prior to surgery to limit inferior outcomes following arthroscopic repair of rotator cuff tears.

## References

[1] Tashjian RZ (2012). Epidemiology, natural history, and indications for treatment of rotator cuff tears. Clin Sports Med.

[2] Brogan DM, Carofino BC, Kircher MF, Spinner RJ, Elhassan BT, Bishop AT, Shin AY (2014). Prevalence of rotator cuff tears in adults with traumatic brachial plexus injuries. J Bone Joint Surg Am.

[3] Teunis T, Lubberts B, Reilly BT, Ring D (2014). A systematic review and pooled analysis of the prevalence of rotator cuff disease with increasing age. J Shoulder Elbow Surg.

[4] Minagawa H, Yamamoto N, Abe H (2013). Prevalence of symptomatic and asymptomatic rotator cuff tears in the general population: From mass-screening in one village. J Orthop.

[5] Yamamoto A, Takagishi K, Osawa T, Yanagawa T, Nakajima D, Shitara H, Kobayashi T (2010). Prevalence and risk factors of a rotator cuff tear in the general population. J Shoulder Elbow Surg.

[6] Tempelhof S, Rupp S, Seil R (1999). Age-related prevalence of rotator cuff tears in asymptomatic shoulders. J Shoulder Elbow Surg.

[7] Hinsley H, Ganderton C, Arden NK, Carr AJ (2022). Prevalence of rotator cuff tendon tears and symptoms in a Chingford general population cohort, and the resultant impact on UK health services: a cross-sectional observational study. BMJ Open.

[8] Dickinson RN, Kuhn JE (2023). Nonoperative treatment of rotator cuff tears. Phys Med Rehabil Clin N Am.

[9] Mancini MR, Horinek JL, Phillips CJ, Denard PJ (2023). Arthroscopic rotator cuff repair: a review of surgical techniques and outcomes. Clin Sports Med.

[10] Fermont AJ, Wolterbeek N, Wessel RN, Baeyens JP, de Bie RA (2014). Prognostic factors for successful recovery after arthroscopic rotator cuff repair: a systematic literature review. J Orthop Sports Phys Ther.

[11] Saccomanno MF, Sircana G, Cazzato G, Donati F, Randelli P, Milano G (2016). Prognostic factors influencing the outcome of rotator cuff repair: a systematic review. Knee Surg Sports Traumatol Arthrosc.

[12] Coronado RA, Seitz AL, Pelote E, Archer KR, Jain NB (2018). Are psychosocial factors associated with patient-reported outcome measures in patients with rotator cuff tears? A systematic review. Clin Orthop Relat Res.

[13] Kennedy P, Joshi R, Dhawan A (2019). The effect of psychosocial factors on outcomes in patients with rotator cuff tears: a systematic review. Arthroscopy.

[14] Verhiel SH, Greenberg J, Zale EL, Chen NC, Ring DC, Vranceanu AM (2019). What role does positive psychology play in understanding pain intensity and disability among patients with hand and upper extremity conditions?. Clin Orthop Relat Res.

[15] Bumberger A, Borst K, Hobusch GM (2021). Higher patient knowledge and resilience improve the functional outcome of primary total knee arthroplasty. Wien Klin Wochenschr.

[16] DiSilvestro KJ, Bond D, Alsoof D (2022). Preoperative resilience and early postoperative outcomes following lumbar spinal fusion. World Neurosurg.

[17] Hoch C, Pire J, Scott DJ, Gross CE (2022). The influence of pain and resiliency on foot and ankle surgery outcomes. Foot Ankle Orthop.

[18] McLaren S, Sims L, Cheng Y, Khan R, Sauder D (2021). The effects of patient resilience and catastrophizing on carpal tunnel surgical outcomes. J Hand Surg Glob Online.

[19] Meade M, Fliegel B, Szukics P, Ford E, Pontes M, McMillan S (2023). Patients with low resilience scores have significantly worse postoperative outcomes after anterior cruciate ligament reconstruction than patients with normal or high resilience scores. Arthrosc Sports Med Rehabil.

[20] Tokish JM, Kissenberth MJ, Tolan SJ (2017). Resilience correlates with outcomes after total shoulder arthroplasty. J Shoulder Elbow Surg.

[21] Glogovac G, Schumaier AP, Kennedy ME, Schramm VT, Wells J, Hasselfeld KA, Grawe BM (2019). Narcotic use and resiliency scores do not predict changes in sleep quality 6 months after arthroscopic rotator cuff repair. Orthop J Sports Med.

[22] Hines AC, Pill SG, Boes N (2022). Mental health status, not resilience, influences functional recovery after arthroscopic rotator cuff repairs. J Shoulder Elbow Surg.

[23] Petrie KA, Lowenstein NA, Collins JE, Matzkin EG (2024). Increased patient resilience scores are related to positive postoperative outcomes in rotator cuff repairs. J Shoulder Elbow Surg.

[24] Porter A, Hill MA, Harm R, Greiwe RM (2021). Resiliency influences postoperative outcomes following rotator cuff repair. J Shoulder Elbow Surg.

[25] Tracy ST, Werner BC, Phillips CJ, Pasqualini I, Ardebol J, Denard PJ (2023). Low resilience is associated with decreased patient-reported outcomes following arthroscopic rotator cuff repair. J Shoulder Elbow Surg.

[26] Wilson CD, Welling BD, Hammonds KA, Robin BN (2022). Impact of patient resilience on early recovery from rotator cuff repair. Shoulder Elbow.

[27] Wilson CD, Villamaria LJ, Welling BD, Hammonds KA, Robin BN (2023). Resilience correlates with patient-reported outcome measures at a minimum of 2 years after arthroscopic rotator cuff repair. Shoulder Elbow.

[28] Longo UG, Candela V, De Salvatore S (2021). Arthroscopic rotator cuff repair improves sleep disturbance and quality of life: a prospective study. Int J Environ Res Public Health.

[29] Kunze KN, Movasagghi K, Rossi DM, Polce EM, Cohn MR, Karhade AV, Chahla J (2020). Systematic review of sleep quality before and after arthroscopic rotator cuff repair: are improvements experienced and maintained?. Orthop J Sports Med.

[30] Haffar A, Bryan S, Harwood M, Lonner JH (2021). Patient Resilience Has Moderate Correlation With Functional Outcomes, but Not Satisfaction, After Primary Unilateral Total Knee Arthroplasty. Arthroplast Today.

[31] Lynskey SJ, Ling F, Greenberg AM, Penny-Dimri JC, Sutherland AG (2021). The influence of patient resilience and health status on satisfaction after total hip and knee arthroplasty. Surgeon.

[32] Magaldi RJ, Staff I, Stovall AE, Stohler SA, Lewis CG (2019). Impact of resilience on outcomes of total knee arthroplasty. J Arthroplasty.

[33] March MK, Harmer AR, Thomas B, Maitland A, Black D, Dennis S (2022). Does resilience predict hospital length of stay after total knee arthroplasty? A prospective observational cohort study. Arthroplasty.

[34] Trinh JQ, Carender CN, An Q, Noiseux NO, Otero JE, Brown TS (2021). Resilience and depression influence clinical outcomes following primary total joint arthroplasty. J Arthroplasty.

[35] Zabat MA, Lygrisse KA, Sicat CS, Pope C, Schwarzkopf R, Slover JD (2022). The impact of patient resilience on discharge after total hip arthroplasty. J Arthroplasty.

[36] Joyce S, Shand F, Tighe J, Laurent SJ, Bryant RA, Harvey SB (2018). Road to resilience: a systematic review and meta-analysis of resilience training programmes and interventions. BMJ Open.

[37] Nakao M, Shirotsuki K, Sugaya N (2021). Cognitive-behavioral therapy for management of mental health and stress-related disorders: recent advances in techniques and technologies. Biopsychosoc Med.

[38] Johnson DC, Thom NJ, Stanley EA (2014). Modifying resilience mechanisms in at-risk individuals: a controlled study of mindfulness training in Marines preparing for deployment. Am J Psychiatry.

[39] Hanley AW, Gililland J, Erickson J, Pelt C, Peters C, Rojas J, Garland EL (2021). Brief preoperative mind-body therapies for total joint arthroplasty patients: a randomized controlled trial. Pain.

